# The Perceptions and Experience of Surgical Trainees Related to Patient Safety Improvement and Incident Reporting: Structured Interviews With 612 Surgical Trainees

**DOI:** 10.7759/cureus.20371

**Published:** 2021-12-12

**Authors:** Hamish Jeffrey, Thomas Samuel, Edward Hayter, Jonas Schwenck, Oliver T Clough, Raymond E Anakwe

**Affiliations:** 1 Trauma and Orthopaedics, Imperial College Healthcare NHS Trust, London, GBR; 2 Trauma and Orthopaedics, Imperial College London, London, GBR

**Keywords:** incident reporting, patient safety improvement, surgical trainee, invasive procedures, patient safety

## Abstract

Background

We undertook a prospective qualitative study to ascertain the perceptions and experience of trainee doctors in the first two years of formal core surgical training related to patient safety improvement and incident reporting. We sought to explore the beliefs, knowledge and opinions of core surgical trainees related to patient safety improvement, their understanding of existing patient safety initiatives and their experience and attitudes to incident reporting.

Methods

We identified 1133 doctors in formal core surgical training posts in the United Kingdom at this time, and we contacted these doctors to invite them to participate in our study. We received responses from 687 (60.6%) core surgical trainees, and 612 trainees (54%) agreed to participate.

The study participants underwent an interview using structured questions asked by trained assessors with an opportunity to explore any particular themes identified by the trainee in more detail. Qualitative data related to the knowledge, experience and perceptions of safety improvement and incident reporting were collected.

Results

Overall, 163 surgical trainees (26.6%) reported that they felt that they could impact patient safety positively. A total of 222 trainees (36.3%) had been involved in or witnessed an adverse patient safety event, while 509 trainees (83.2%) reported that they had witnessed a ‘near-miss’ event. Only 81 trainees (13.2%) had submitted a patient safety report at some point in their career. In addition, 536 trainees (87.6%) reported that they considered a patient safety or incident report to be ‘negative’ or ‘seriously negative’ and that they would be discouraged from making these because of the negative connotations associated with them.

Of the 81 core surgical trainees who had submitted a patient safety report, only nine trainees (11.1%) reported that they had received a meaningful reply and update following their report. Several themes were identified during the interviews in response to open questions. These included a perception that patient safety improvement is the responsibility of senior surgeons and managers and that surgical trainees did not feel empowered to influence patient safety improvement. Trainees expressed the view that incident reporting reflected negatively on clinicians and the standard of care provided, and there were reports of culture based on blame and the fear of litigation or complaints. Surgical trainees did not feel that incident reporting was an effective tool for patient safety improvement, and those trainees who had made patient safety reports felt that these did not result in change and that they often received no feedback.

Conclusions

Core surgical trainees report that they are not well engaged in patient safety improvement and that their perceptions and experience of incident reporting are not positive. This represents a missed opportunity. We suggest that in order to recruit the surgical workforce to the improvement work and learning associated with patient safety, opportunities for focused education, training and culture change are needed from the early years of surgical training. In addition, improvements to the processes and systems that allow trainees to engage with safety improvement are needed in order to make these more user-friendly and accessible.

## Introduction

Patient safety improvement has traditionally been focused on the technical aspects of quality and the delivery of care. It is increasingly recognised that cultural and human factors are important contributors to patient safety improvement and that elements of both can be taught and role modelled. Adverse patient safety incidents have the potential to cause serious and lasting harm to patients. Many healthcare organisations have placed a focus on systems and processes to reduce the potential for human error and patient harm, particularly where this is related to surgery and procedural interventions. There is good evidence that a focus on culture and human factors can improve patient safety and that these are most effective when they address the whole organisation [1,2].

Surgical trainees are responsible for a large number of patient contacts encompassing the preoperative, perioperative and postoperative phases of care. Their interactions with patients, record keeping, handovers of care, communication and technical preparation and skills all contribute to the safety of each phase of care. Direct care is often delegated to surgical trainees who, by virtue of their position, are close enough to the everyday processes of care. This provides them with an excellent appreciation of patient safety risks, any potential solutions or workarounds and how these risks and problems could be resolved. For example, in a busy day surgery unit where the printer for blood labels is not working, having to take blood samples from patients and then transport them to another ward to print the labels can significantly increase the risk of mislabelling samples and attributing blood results to the wrong patient. This simple process risk may not be apparent to anyone other than the junior doctors who are delegated the task of taking patient blood samples, but they may not feel empowered or engaged enough to identify, own and report the risk so that it can be resolved.

We undertook a prospective qualitative interview-based study to ascertain the perceptions and experience of trainee doctors undertaking core surgical training. We aimed to explore the beliefs, knowledge and opinions of these doctors in relation to patient safety improvement, their understanding of existing patient safety initiatives and their experience and attitudes to incident reporting.

## Materials and methods

We hypothesised that core surgical trainees were not actively engaged in patient safety improvement work, did not feel that they could influence this and would report neutral or negative views and experience of incident reporting.

The study was designed as a prospective qualitative study using data from interviews with core surgical trainees conducted over a video platform. The interview consisted of seven structured questions followed by a themed discussion based around four open-ended questions. Surgical trainees were identified by contacting each of the training deaneries across the United Kingdom to identify all core surgical trainees in post as of January 1, 2021. Each interview was conducted by one of the authors. Questions and interview structure were agreed upon by all interviewers in a preliminary training meeting to ensure consistency. The open-ended questions were used to identify any recurring themes related to the study hypothesis.

We identified 1133 doctors in core surgical posts as of January 1, 2021. Of these, 687 doctors (60.6%) responded to our request, and 612 doctors (54%) agreed to participate in the study. Participation was voluntary, and responses were anonymised. Study participants were interviewed over the Zoom^TM ^video platform, and the interview was open-ended and untimed.

This study was approved by the Institutional Clinical Audit and Effectiveness Group (approval CAEG/SCC/2021/raF1).

Statistical analysis

Data were analysed using IBM SPSS® Statistics Version 26.0. A simple descriptive analysis only was required. Numerical scores were expressed as means with ranges.

## Results

We recruited 612 study participants, who made up 54% of core surgical trainees across the United Kingdom. A total of 401 trainees (65.5%) were men, and the mean age was 31 (range: 24-34) years. In addition, 371 trainees (60.6%) were in their second year of the two-year core surgical programme.

The questions asked in the structured interview and the responses from the core surgical trainees are summarised in Table 1.

**Table 1:** Structured Interview Questions and Core Surgical Trainee Responses

Structured Interview Questions	Core Surgical Trainee Responses	Number (%)
How involved or engaged are you with patient safety and safety improvement?	Heavily involved	2 (0.3%)
	Involved	40 (6.5%)
	Aware of ongoing work	350 (57.2%)
	Not involved	220 (35.9%)
	Not interested	0 (0%)
In your workplace, who is responsible for patient safety? (Choose any/all relevant options.)	Consultants, managers, junior doctors and nurses	165 (10.6%)
	Consultants and managers	364 (59.5%)
	Consultants	68 (11.1%)
	Junior doctors and nurses	15 (2.5%)
How equipped do you feel to positively influence patient safety and safety improvement?	Very well equipped	32 (5.2%)
	Well equipped	131 (21.4%)
	Neutral	324 (52.9%)
	Poorly equipped	95 (15.5%)
	Very poorly equipped	30 (4.9%)
Have you ever reported a patient safety risk or incident?	Yes	81 (13.2%)
	No	531 (86.8%)
Have you been involved in or witnessed a patient safety risk or incident at work?	Yes	222 (36.3%)
	No	390 (63.7%)
Have you been involved in or witnessed a ‘near-miss’ incident at work?	Yes	509 (83.2%)
	No	103 (16.8%)
Have you ever had any training in patient safety improvement or incident reporting?	Yes	397 (64.9%)
	No	215 (35.1%)
Themed Questions		
What do you think are the main risks to patient safety in the operating theatre and when undertaking procedures? How can we reduce errors in this environment?		
Why don’t junior doctors report more patient safety incidents?		
How can we engage and recruit surgical trainees to be more involved in patient safety improvement?		
What are your perceptions of incident reporting as a tool for patient safety improvement?		

Only 42 trainees (6.9%) considered that they were involved or engaged in patient safety and safety improvement. The remainder considered that they were not involved (220 trainees, 35.9%) or were aware of ongoing work (350 trainees, 57.2%). One hundred sixty-five trainees (27%) identified patient safety as the responsibility of all consultants, junior doctors, nurses and managers. Most trainees identified this to be the responsibility of consultants and managers (364 trainees, 59.5%) or consultants alone (68 trainees, 11.1%). In response to the open questions, almost all trainees felt that their focus was on performance and efficiency, completing surgical or procedural cases, efficiently reviewing and treating patients and acquiring training and skills at the same time. Patient safety and safety improvement were acknowledged as important by most trainees but were generally not thought to be a core part of their job or to be a major part of how they are assessed.

On the whole, trainees were positive about their ability to positively influence patient safety and safety improvement, although 531 trainees (86.8%) reported that they had never reported a patient safety risk or incident before. Five hundred nine trainees (83.2%) reported that they had witnessed or been involved in a ‘near-miss’ safety incident. Three hundred ninety-seven trainees (64.9%) reported that they had received some training in patient safety improvement and incident reporting.

The answers to the themed open questions identified that trainees believed that the main risks to patient safety in invasive procedures and surgery centred on technical ability or errors, issues with consent, wrong-site surgery, unexpected complications and anatomical variation. The initial discussion focused on technical factors. Several trainees talked about the benefit of having standardized routines and processes that are understood by all and the importance of checks before key steps in the surgery or procedure. Training, standardization of processes, a ‘surgical pause’ before a procedure is started, checklists, two-person checks and supervision were all identified as ways of reducing and mitigating the risks to patient safety in the operating theatre and procedural environment. The themes of culture, behaviours and role modelling were raised by trainees who made the point that the culture and atmosphere in which surgery or a procedure is being performed can impact the ability of the surgeon to function and of the whole team to contribute to a safe procedural environment.

Trainees felt that there were several barriers to engaging junior doctors in incident reporting. They reported that they did not feel that it was an effective method to drive safety improvement and that, when they did report a safety incident, they often could not identify any change or improvement and received very little feedback. Several trainees reflected that they would avoid submitting a patient safety report because it might reflect badly on their senior team or supervisor and also that it imposed an additional administrative burden on busy clinicians. Those core surgical trainees who had reported a patient safety incident felt that these were often used to apportion blame when processes or procedures went wrong. They described the use of incident reporting to highlight or ‘punish’ perceived poor behaviours in the operating theatre, failures of efficiency and performance where surgical cases or procedures were cancelled or where incident reporting was used to escalate a ‘failure to act’. In this regard, the link with patient safety and improvement was lost, and almost all of the surgical trainees interviewed felt that incident reporting had negative connotations and was not used as a positive tool.

Trainees broadly felt that the systems to allow incident reporting were not easily accessible. They were often accessed via the hospital intranet, which could only be accessed via a hospital desktop computer. Many of the trainees understood that, following an incident report, there would be an investigation, but they were not entirely clear about who undertook this, how it happened, their role in this and how the results of the investigation would be communicated. Almost all trainees felt that the systems and processes for incident reporting could be made more user-friendly and accessible.

## Discussion

Healthcare organisations continue to report adverse patient safety incidents despite the efforts and interventions that have been put in place to prioritize patient safety and safety improvement [3-6]. These increasing numbers of incidents include ‘never events’, and these are often themed around surgery and procedural interventions. This is despite well-established safety interventions such as the World Health Organisation (WHO) surgical safety checklist [7]. This may represent an improved culture of incident reporting rather than any true deterioration in patient safety. It does imply that there continue to be episodes of avoidable harm for patients.

Our study shows that core surgical trainees are often aware of ongoing patient safety improvement work, but they are not well engaged in these projects and efforts. This would seem to be a missed opportunity considering the volume of clinical contacts and interventions that surgical trainees have with patients and the associated opportunities to actively improve and advocate for patient safety.

The negative perceptions of these trainees with respect to incident reporting are also concerning, and trainees reported that these views are reinforced by the observed values and behaviours of senior colleagues and the multidisciplinary team. This suggests that a larger cultural and whole workforce approach is needed to make meaningful change and improvements in the attitudes to and perceptions of incident reporting as a tool for safety improvement. There is good evidence that increased incident reporting and reporting across different professional groups is a marker of an organisation with strong safety culture and an approach of learning, openness and ‘just culture’ when adverse events do happen [8,9].

The United Kingdom national patient strategy has resulted in the first multidisciplinary patient safety syllabus, which has recently been published and is now being implemented across the National Health Service [10]. This provides a comprehensive framework for training healthcare staff in risk, systems and organisational culture with a focus on patient safety and safety improvement. Our work supports the assertion that in addition to this additional training, education and the culture change they are designed to produce, further work is needed to make the systems and processes that allow surgical trainees to engage with incident reporting and patient safety improvement more accessible and user-friendly.

## Conclusions

Core surgical trainees report that they are not actively engaged in patient safety improvement and that their perceptions and experience of incident reporting are not positive. This represents a missed opportunity. We suggest that in order to recruit the surgical workforce to become actively involved in patient safety improvement work, opportunities for focused education, training and the development of a strong safety culture are needed from the early years of surgical training.

The volume of their clinical contacts, the fact that they interact with patients through the pre-, peri- and postoperative phases of care and their ubiquitous presence in direct patient-facing roles make surgical trainees ideal advocates for safety improvement particularly in the surgical, procedural and perioperative environment. The new national patient safety syllabus presents an opportunity to partly address current shortcomings with education and training, but there are other aspects of organisational culture, systems and processes of reporting as well as attitudes to incident reporting that also need to be addressed in order to make this effective.
